# Selenium Biofortification and Interaction With Other Elements in Plants: A Review

**DOI:** 10.3389/fpls.2020.586421

**Published:** 2020-11-05

**Authors:** Xinbin Zhou, Jing Yang, Herbert J. Kronzucker, Weiming Shi

**Affiliations:** ^1^College of Resources and Environment, Southwest University, Chongqing, China; ^2^Faculty of Land and Food Systems, University of British Columbia, Vancouver, BC, Canada; ^3^State Key Laboratory of Soil and Sustainable Agriculture, Institute of Soil Science, Chinese Academy of Sciences, Nanjing, China

**Keywords:** Se, biofortification, nutrient elements, heavy metal, interaction effect

## Abstract

Selenium (Se) is an essential element for humans and animals and its deficiency in the diet is a global problem. Crop plants are the main source of Se for consumers. Therefore, there is much interest in understanding the factors that govern the accumulation and distribution of Se in the tissues of crop plants and the mechanisms of interaction of Se absorption and accumulation with other elements, especially with a view toward optimizing Se biofortification. An ideal crop for human consumption is rich in essential nutrient elements such as Se, while showing reduced accumulation of toxic elements in its edible parts. This review focuses on (a) summarizing the nutritional functions of Se and the current understanding of Se uptake by plant roots, translocation of Se from roots to shoots, and accumulation of Se in grains; and (b) discussing the influence of nitrogen (N), phosphorus (P), and sulfur (S) on the biofortification of Se. In addition, we discuss interactions of Se with major toxicant metals (Hg, As, and Cd) frequently present in soil. We highlight key challenges in the quest to improve Se biofortification, with a focus on both agronomic practice and human health.

## Introduction

Se is an essential trace element for human and animal health, where it can be covalently incorporated into amino acids, chiefly selenocysteine (SeCys) and selenomethionine (SeMet), and acts as a cofactor for antioxidant enzymes such as glutathione peroxidase, and, in these chemical forms, is involved in the maintenance of the immune system, regulation of thyroid function, brain cognitive function, general antioxidant and detoxification capacity, and anti-cancer and anti-viral effects have also been documented (Hatfield et al., 2014; Ullah et al., 2019). Se deficiency in the human body can lead to serious medical complications, including cataracts, endothelial dysfunction and cardiovascular disease, cardiomyopathy, osteochondropathy, poor immune function, cognitive decline, and even cancer (Natasha et al., 2018; Newman et al., 2019). However, more than about 15% of the world’s population suffer from Se deficiency, including in many regions of China, Oceania, Africa, and Europe (Tan et al., 2016; Schiavon and Pilon-Smits, 2017; Dinh et al., 2018). Plants are the major dietary source of Se for humans, but Se levels in cereals such as rice and wheat are generally low (Zhu et al., 2009; Williams et al., 2009). Se deficiency is a long-standing public health problem (Gao et al., 2018; D’Amato et al., 2018). In China, low Se concentrations in staple food has been linked to decreased Se concentrations in hair (Li et al., 2014; Dinh et al., 2018). Se deficiency can be countered with Se-enriched food. Biofortification of crops is an economically viable and safe agricultural technique, and can be aimed at increasing Se concentration in the edible parts of crops, thereby overcoming Se deficiency in the diet (White and Broadley, 2009).

In plants, moderate Se is not considered essential but acts as a clearly beneficial element and can promote plant growth (Pilon-Smits et al., 2017; Jia et al., 2018). It has been shown to be involved in the regulation of photosynthesis and respiration, stress resistance, antioxidant capacity, abiotic stress tolerance, and alleviation of heavy metal stresses (Ulhassan et al., 2019; Dai et al., 2019; Duan et al., 2019). It is important to point out that there is a narrow range of Se accumulation in tissues within which Se deficiency transitions to toxicity in humans, animals, and plants. Excessive amounts of selenium can cause toxicity to all organisms. This article focuses on the range of Se doses in Se applications below the toxicity threshold.

In recent years, pollution by heavy metals, especially As, Hg, and Cd, which possess relatively high mobility in agricultural soil, has caused widespread concern. These metals are readily absorbed by most plants and pose a potential health risk to livestock animals and humans as they enter the food chain (Zhao F. et al., 2010). In the past 10 years, great progress has been made in reducing heavy metal absorption and accumulation in grains by virtue of Se soil amendments (Zhang H. et al., 2014; Tran et al., 2018a; Camara et al., 2019; Li et al., 2019a).

To sum up, Se is of great nutritional significance in humans and of significant importance in reducing the bioconcentration of heavy metals in the food chain (Dinh et al., 2019). An ideal crop is one with high bioavailability of essential elements such as Se, but with reduced accumulation of metals such as Cd, As, and Hg in the edible parts (Huang S. et al., 2020). Therefore, in this review, we provide a summary of our current understanding of the mechanisms of Se uptake by plants, Se transport between root to shoot, within-plant Se distribution between organs and sequestration within edible parts, and of the principal interaction mechanisms with macronutrient elements and heavy metals, with an emphasis on the implications for Se biofortification and reductions in the toxic metal load of crop plants.

## Pathways of Se Uptake From the Soil to Plant Roots

Plant roots can absorb inorganic Se as Se(VI) (selenate), Se(IV) (selenite), elemental Se(0) and organic Se species, such as SeCys and SeMet, but cannot absorb Se(-II) selenide (Wu et al., 2015; White, 2016). There are different forms of Se in soils as a function of pH and Eh. In oxic soils (pH + pE > 15), selenate is the most abundant form, while, in anaerobic soils with a neutral to acidic pH (pH + pE = 7.5–15), selenite (SeO_3_^2–^; HSeO_3_^–^; H_2_SeO_3_) is the most abundant form (White, 2018). Nutrients absorbed by plants mainly come from the rhizosphere, and the local conditions in the rhizosphere can influence the bioavailability of Se to plants (Zhou et al., 2007; Chen et al., 2010). Plant roots selectively take up different forms of Se by different mechanisms, and the mobility and metabolism of different forms of Se in plants lead to different seleno-compounds.

### Selenate Uptake

Se and sulfur (S) are both group-16 “chalcogens” in the periodic table, meaning they have similar ionic radii, covalent radii, and chemical properties (White, 2016). Indeed, selenate enters plant roots using sulfate transporters (Gupta and Gupta, 2017). In *Arabidopsis thaliana*, SULTR1;1 and SULTR1;2 are the two high-affinity sulfate transporters resident in the plasma membrane that can facilitate the transport of Se(VI) (Schiavon et al., 2015). Different plant roots have different transporters for Se(VI) absorption. At present, no specific Se(VI) transporter has been found in any organism, but the results of bioinformatics analysis suggest that specific Se(VI) transporters may exist in Se hyperaccumulators (Harris et al., 2014). However, no candidate for a specific Se(VI) transporter has, to date, been identified.

### Selenite Uptake

**FIGURE 1:**
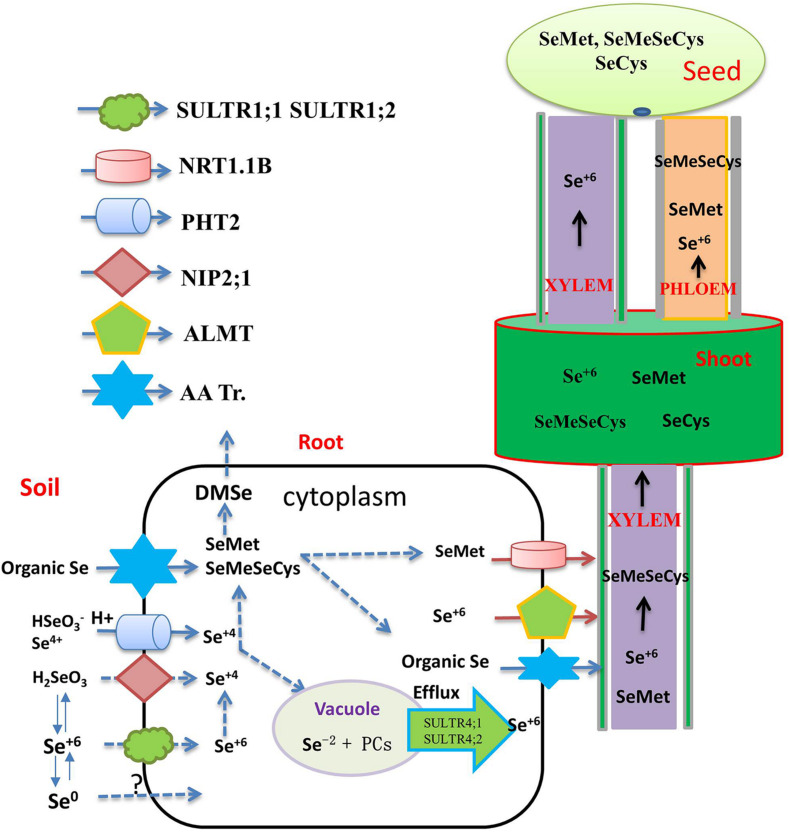
Flow diagram representing the transporters involved in the uptake, translocation, and accumulation of different selenium species through xylem and phloem to the grain. The main transporters involved in inorganic Se uptake by plants (SULTR1;1, SULTR1;2 for selenate, NIP2;1, PT2 and PT8 for selenite, AA Tr. For amino acids). Organic Se forms are transported into the xylem via amino acid permeases (AA Tr.) and delivered to the shoots. Selenate is the major Se species present in the xylem and loaded into the xylem by SULTR2;1. Organic-Se compounds are transported into the seed via the phloem, while selenate is transported via both xylem and phloem. The translocation of SeMet to the seeds is enhanced by overexpression of the NRT1;1B transporter.

Selenite is a weak acid, which can exist in different forms under varying pH and Eh (Zhou et al., 2018; White, 2018). Different absorption mechanisms for Se(VI) appear to be in operation under different pH regimes. Zhao X. et al. (2010) found that, under acidic conditions, the rice silicon transporter OsNIP2;1 absorbs H_2_SeO_3_. In slightly acidic paddy soils, selenite prevails in the form of HSeO_3_^–^, which is structurally similar to H_2_PO_4_^–^. In keeping with this, a phosphate transporter OsPT2 has been found to catalyze the transport of HSeO_3_^–^ (Zhang L. et al., 2014; Figure 1). Song et al. (2017) showed, in transgenic tobacco lines, that the absorption of phosphate and Se accumulation in shoots is improved by over-expression of *OsPT8*, but it remains unclear whether this is a more universal transport relationship between the two elements in plants. It emerges that several parallel selenite absorption pathways exist in plants, only some of which are related to P absorption, and very few genes related to Se absorption in plant have hitherto been explored, highlighting the need for much more concentrated research on the molecular mechanism of Se uptake, transport, and metabolism (Table 1 and Figure 1).

**TABLE 1:** The main genes involved in Se uptake, transport, and metabolism in plant.

Function	Gene category	Gene name	References
**Se uptake transport protein**			
H_2_SeO_3_ uptake by root	Silicon influx transporter	*OsNIP2;1*	Zhao X. et al., 2010
HSeO_3_^–^ uptake by root	Phosphate transporter	*OsPT2*	Zhang L. et al., 2014
Se(VI) uptake by root	Sulfate transporter	*AtSULTR1;2*	Ei et al., 2007
Selenite uptake and transport by root	Phosphate transporter	*OsPT8*	Song et al., 2017
Selenite and Pi uptake in root of tobacco	Phosphate transporter	*NtPT2*	Jia et al., 2018
SeMet translocation from roots to shoots and grain	Rice peptide transporter (PTR)	*NRT1.1B*	Zhang et al., 2019
**Key enzymes of Se metabolism pathway**			
Catalyze Se(VI) into APSe	ATP sulfurylase	*APS*	Sors et al., 2005
Catalyze APSe into Se(IV)	APS reductase	*APR*	Sors et al., 2005
Se(IV) reduction to Se(II-)	Sulfite reductase enzyme	*SiR*	White, 2018
Catalyze Se(IV) into SeCys	Selenomethyltransferase	*SMT*	Chauhan et al., 2019
Catalyze the synthesis of other organic species from Se-Cys	Cystathionine-γ-synthase	CGS	White, 2018

### Organic Se Species Uptake

Compared with studies on inorganic Se, there is comparatively much less research to date on the absorption and transport of organic forms of Se by plants. Kikkert and Berkelaar (2013) showed that SeCys and SeMet were both taken up at rates that were some 20-fold higher than those observed for selenate or selenite. Se-amino acids, in particular, are likely to enter plant cells via amino acid transporters (AA Tr.) (Figure 1; Lima et al., 2018; Schiavon et al., 2020). It has therefore been reasoned that SeCys and SeMet can be taken up by this amino acid transporter as well. As there are many classes of amino acid transporters, it is reasonable to hypothesize that other amino acid transporters will also be involved in the absorption of organic forms of Se, but work in this area remains scant.

## Se Translocation From Root to Shoot

Inorganic forms of Se absorbed by plants are transported from the root to the shoot through the xylem. The transport process depends on the form of externally supplied Se. Se(VI) can be easily absorbed and transported by the xylem, and then distributed further to reproductive organs by the phloem (Carey et al., 2012). Members of the ALMT transporter family, such as AtALMT12, are thought to load Se(VI) into the xylem sap, and Se(VI) is the major Se species present in the xylem, although small amounts of SeMet and SeOMet are also found (White, 2018; Figure 1).

In most plants, Se(VI) enters mesophyll cells through the SULTR transporter, and its accumulation then occurs in the vacuole. The transporter responsible for the entry of Se(VI) across the tonoplast into the vacuole remains undefined, but homologs of AtSULTR4;1 and AtSLUTR4;2 have been proposed to act as efflux transporters for Se(VI) exiting vacuoles (White, 2016). Se(VI) is reduced to Se(IV) in plant leaves, and, once reduced, Se is then incorporated into organic Se compounds and distributed to other tissues. Carey et al. (2012) suggested that Se(VI), SeMet, and Se-methylselenocysteine (MeSeCys) transported via the xylem are then readily redistributed to sink organs via the phloem. In *Arabidopsis*, AtSULTR1;3 and AtSULTR2;2 seem to promote Se(VI) to enter the phloem, and the expression of genes encoding AtSULTR1;3 and AtSULTR2;2 is upregulated by Se accumulation (Wang et al., 2018).

Following Se(IV) absorption by roots, rapid assimilation into organic forms, including SeMet and MeSeCys, can occur. These seleno amino acids mainly accumulate in the root, with only a small portion transferred to the shoot, rendering the root system the site of higher bioconcentration of the element (Winkel et al., 2015), a potential impediment to Se biofortification of crop plants, as most edible parts are above-ground.

The transport of ions or molecules to the aboveground plant tissues depends on the xylem loading rate and the rate of transpiration (Renkema et al., 2012). Kikkert and Berkelaar (2013) verified that different Se species are transported at significantly different rates in canola and wheat, in the order: Se(VI) > SeMet > Se(IV)/SeCys. This primary Se absorption difference is due to regulation of transporters resident in the plasma membrane of root cells. Recently, Liang et al. (2019) demonstrated that a low concentration of Se in the supply medium induces the expression of *OsNIP 2;1*, which may serve as a strong positive regulator of Se transport in rice.

## Long-Distance Translocation and Se Accumulation in Aboveground Tissues of Plants

Long-distance Se transport is important for regulating Se accumulation and increasing Se concentration in particular in crop-plant tissues that are most often consumed by livestock and by humans. Se speciation in edible parts differs among plants. For example, Se in rice grains is mainly in the form of SeMet, followed by SeMeSeCys, and SeCys (Carey et al., 2012). MeSeSeCys, Se(VI), and Se(IV) are the main Se compounds in the aboveground tissues of *broccoli* (Wu et al., 2015). In staple food crops such as the grain crops wheat, barley, and rye, SeMet is the main Se compound (Poblaciones et al., 2014), while in rice grains, SeMet is the main Se species, accounting for 82.9% of total Se, followed by MeSeCys accounting for 6.2% of total Se, and SeCys accounting for 2.8–6.3% of total Se (Sun et al., 2010). Carey et al. (2012) have suggested that organic Se (SeMet and SeMeSeCys) is transported to the grain exclusively via the phloem, while inorganic Se is transported to the grain by both phloem and xylem. Different Se forms have different nutritional value to humans, among which SeMeSeCys has superior anti-cancer properties (Roman et al., 2014). Thus, with a view to human health, increasing the concentration of SeMeSeCys in the edible parts of crops by means of biofortification is a major goal. With the refinement of molecular biology techniques, and on the basis of studying the mechanisms of Se transport and metabolism, it has become possible to achieve overexpression of target genes related to Se enrichment in specific plant tissues, such as grains, so as to improve the Se concentration of crops. Zhou et al. (2017c) found that Se-enriched Xiushui 48 rice can transport more organic Se from flag leaves to grains than a non-Se-enriched variety, explaining the pronounced difference between these rice varieties in their grain Se concentration. It is still unknown whether Se transporter protein activity and expression levels in Se-rich varieties are higher than those in non-Se-rich varieties. NRT1.1B is responsible for transporting SeMet to rice grains, thus improving grain accumulation of Se, and transgenic lines overexpressing *NRT1.1B* have significantly increased grain Se concentration (Zhang et al., 2019; Figure 1). These above studies not only deepen our understanding of Se uptake and translocation mechanisms, but also provide a theoretical basis for breeding Se-enriched crop varieties.

## Metabolism of Se in Plants

Se and S share the same metabolic pathway in plants. Se absorbed by roots is delivered to leaf chloroplasts via the xylem. Once inside chloroplasts, Se(VI) is reduced to adenosine phosphoselenate (APSe) under ATP sulfurylase (APS), and the reduction to Se(IV) is catalyzed by adenosine phosphoselenate reductase (APR), using glutathione (GSH) as the electron donor; this step is the rate-limiting step for Se metabolism into organic components. Se(IV) reduction to Se(II-) is performed by the sulfite reductase enzyme (SiR) (White, 2018). Se(II-) can then react with O-acetylserine (OAS) to form Se-cysteine (SeCys) (White, 2018). Alternatively, SeCys can be formed directly from selenite by the selenomethyltransferase enzyme (SMT) (Chauhan et al., 2019). Further transformation of SeCys into other organic species can occur via cystathionine-γ-synthase (CGS), cystathionine-β-lyase (CβL), SeCys methyl synthase (SMT) methylation, with the products of SeMet and MeSeCys and MeSeMet. SeMet and MeSeCys can then be further metabolized into selenoproteins and volatile Se species such as dimethylselenide (DMeSe) or dimethyldiselenide (DMeDSe) (Gupta and Gupta, 2017; White, 2018).

In recent years, a host of genes coding for Se uptake, transport, and metabolism in plants have been identified (Table 1). Manipulating, and capitalizing on the natural variation in, the expression of these genes will play a vital role in improving the accumulation, tissue-specific distribution, and chemical speciation of Se in plants. Understanding the interplay of the key genes is urgent so as to afford the tools for improvement in the biofortification potential of crops that will benefit animal and human nutrition.

## Se Interactions With Macronutrient Elements (N, P, and S)

### The Role of Nitrogen in the Regulation of Se Bioavailability and Accumulation

N fertilizer plays a central role in increasing crop yield and improving crop quality in all forms of intensive agriculture (Cui et al., 2010). The effect of N on plant Se absorption is bidirectional (Table 2). Reis et al. (2018) demonstrated that, in rice, with increasing N-fertilizer application, the Se concentration in grains increased. Similarly, Chen et al. (2016) showed that high N fertilization can promote uptake and translocation of Se in wheat on low-Se soils. This interaction between N and Se can be explained as follows: (1) N application activates S metabolism and increases S assimilation by increasing O-acetyl serine, a key regulator of S metabolism in cysteine synthesis in higher plants, and then increases the synthesis of cysteine and protein (Kim et al., 1999). Se and S use the same metabolic pathway in plants. Therefore, N application can also promote plant Se absorption, and Se can then be further metabolized into selenoproteins. (2) N fertilizer promotes growth, thereby promoting the absorption of P, K, S, and other mineral elements, including Se, by the root system (Chen et al., 2012); (3) N fertilizer applied to the soil mainly exists in the form of nitrate-N (NO_3_-N), and NO_3_^–^, at high doses, can dislodge Se anions from the surface of soil particles, leading to an increase of SeO_4_^2–^ concentration in soil solution, in turn promoting Se uptake (Dinh et al., 2018). Similarly, Klikocka et al. (2017) observed that Se concentration and uptake in spring wheat grain increased significantly with increasing N-fertilizer application. The results show that N applications are helpful for plants in terms of increasing Se uptake by roots.

**TABLE 2:** Research progress on the effects of nutrient elements on Se accumulation in higher plants.

Added nutrition	Amounts	Crop species	Test results	Se availability	References
N fertilizer	0, 20, 40, 80, 120 kg ha^–1^	Rice	The Se and protein content in rice grain increased with the increase of N application, and the grain Se content increased from 0.03 mg kg^–1^ to 0.35 mg kg^–1^.	Increase	Reis et al., 2018
N fertilizer	100, 200 mg kg^–1^	Wheat	N fertilizer increased the absorption of Se (VI) in wheat root and the migration of Se to the aboveground. With the increase of N fertilizer, the Se content in grain increased by 22.6 and 12.1%, respectively under the treatment of low and high Se.	Increase	Chen et al., 2017
N fertilizer	0, 60 mg kg^–1^	Wheat	N fertilizer increased the content of wheat grain Se.	–	Duncan et al., 2017
N fertilizer	0, 40, 80, 120 kg ha^–1^	Wheat	N fertilizer increased wheat grain Se content by 17.4, 34.8, and 82.6% with the N content of 40, 80, and 120 kg ha^–1^, respectively.	–	Klikocka et al., 2017
P fertilizer	0, 1, 2 g kg^–1^	Wheat	Under Se(IV) treatment, Se content and transfer factor of wheat decreased. P fertilizer reduced the content of soluble and exchangeable Se in the soil and increased iron oxide-bound Se or organic matter-bound Se.	Decrease	Zhang et al., 2017
P fertilizer	0, 1, 2 g kg^–1^	Wheat	Under Se(VI) treatment, Se content and transfer factor of wheat increased. P fertilizer significantly reduced the content of iron oxide-bound and organic matter-bound Se.	Increase	Zhang et al., 2017
P fertilizer	0, 80, 160 mg kg^–1^	Wheat	In the presence of Se(IV), the P fertilizer addition significantly reduced the concentration of various forms of Se.	Decrease	Nie et al., 2020
NH_4_H_2_PO_4_ (NH_4_)_2_HPO_4_	1.96 g kg^–1^ 2.17 g kg^–1^	Wheat	P fertilizer reduced the content of organic matter-bound and Fe and Mn oxide-bound Se, and increased Se content in wheat	Increase	An et al., 2019
P fertilizer	0, 240 mg kg^–1^	Rice	P fertilizer significantly reduced the Se concentrationin rice grain by 15.2%.	Decrease	Shen, 2017
S fertilize	0, 20 mg kg^–1^	Wheat	S fertilizer decreased the content of wheat grain Se.	–	Duncan et al., 2017
S fertilize	0, 50 kg ha^–1^	Wheat	S fertilizer increased the content of wheat grain Se by 20.8%.	–	Klikocka et al., 2017
S fertilize	0, 140 mg kg^–1^	Rice	S fertilizer significantly reduced the Se concentrationin rice grain by 19.1%.	Decrease	Shen, 2017
S fertilize	0, 150, 300 mg kg^–1^	Rape	S application reduced the Se content in rape and promoted the growth of rape. Sulfur application only affected the Se absorption in root, but not the Se transportation. Sulfur application significantly reduced rape seed Se content by28.2% and 40.4% with the sulfur content of 150 mg kg^–1^ and 300 mg kg^–1^ respectively.	Decrease	Liu et al., 2016b

However, N-fertilizer applications can also lead to decreases in Se concentration. Morlon et al. (2006) reported a significant inhibition of Se(IV) uptake by NO_3_-N in the alga *Chlamydomonas reinhardtii*. One mechanism by which such inhibition might occur for higher plants in soils lies in the fact that high doses of N can promote soil acidification (Guo et al., 2010), thereby reducing Se availability by virtue of the formation of less plant-available ferric-iron-selenite complexes (Gupta and Gupta, 2000). Moreover, Xu et al. (2010) demonstrated that anions such as NO_3_^–^, SeO_4_^2–^ or SeO_3_^2–^, can antagonize, or compete with, each other’s absorption individually and collectively. In addition, under low-nitrate conditions, plant Se concentration has been shown to be positively correlated with soil-extractable NO_3_-N concentration, whereas, under high-nitrogen conditions (more than 600 mg kg^–1^), plant Se concentration was negatively correlated with soil-extractable NO_3_-N (Li et al., 2015). The results show that high-N applications can at times reduce Se uptake by plants.

Therefore, N fertilizers can have two-way effects on the bioavailablity of Se, by diverse mechanisms. Clearly, the relationship between tissue Se accumulation in plants and N supply on different soil types and under varying N-nutrient-management regimes in the field needs dedicated examination (Dinh et al., 2018).

### The Role of Phosphorus in the Regulation of Se Bioavailability and Accumulation

Although P and Se are non-congeners, both are absorbed by plants when present in the anionic form in the soil, and they have similar ionic radii and physical and chemical properties. Hence, competition with oxyacid anions in soil is expected to affect the absorption and accumulation of Se by plants. However, current literature reports on the relationship between plant Se uptake and P levels and acquisition have shown conflicting results (Table 2). The hydrogen phosphate ion can significantly reduce Se(IV) absorption from the soil by competition, however, increasing soil-solution Se concentrations, while Se in soil solution is easily absorbed by plants (Lee et al., 2011; Zhou et al., 2015). Zhang et al. (2017) reported that P fertilization together with Se(IV) could significantly reduce Se accumulation in wheat in calcareous soil, but in the case of P fertilization together with Se(VI), there was increased Se accumulation in wheat. Such differences show that the effect of P application on plant Se uptake and accumulation depends on the type of Se speciation in the soil (Zhang L. et al., 2014). The reason is that P improves bioavailability of Se(VI), but P application lowers soil pH and activates soil iron ions, and iron has a high affinity for Se, converting soluble Se in the soil into iron oxide Se and organic Se. These two forms of Se are difficult for plants to absorb, i.e., Se(IV) absorption by crops is reduced under combined applications of P fertilizer and Se(IV) (Szlachta et al., 2012). On the other hand, P fertilizer activated Fe-oxide-bound Se and organic-matter-bound Se in calcareous soil, and increased bioavailability and Se concentration in wheat (Zhang et al., 2017). In addition, the influence of P application on Se uptake and accumulation in plants depends on the rate of the P application (Liu et al., 2018; Li et al., 2019b). Nie et al. (2020) also demonstrated that all Se fractions (total, organic, inorganic) in the grain of winter wheat were significantly decreased under increasing P application rates. Therefore, in agricultural production, it is very important to avoid excessive use of P fertilizer, so that a balance is achieved between higher yield and appropriate grain Se concentration. Proper P management is crucial to grain Se concentration control.

### The Role of Sulfur in the Regulation of Se Bioavailability and Accumulation

Sulfur and Se are elements of the same group in the periodic table, the “chalkogens.” Therefore, S and Se have many chemical similarities. Studies have shown that plants absorb and assimilate Se and S using identical pathways (Table 2). Se(VI) as selenate is structurally similar to SO_4_^2–^, and Se(VI) is absorbed via S transporters resident in the root plasma membrane (Terry et al., 2000). S-fertilizer regimes in soils have potential significance in regulating Se bioavailability for many plant species (Duncan et al., 2017). The interaction between S and Se in plants is complex (Table 2). Since the same transporters are shared when plants absorb S and Se(VI), there is often a competitive relationship in the absorption of S and Se. The expression of sulfate transporters will vary depending on crop species and S level. Hence, S fertilization can increase or decrease grain Se concentration, depending on crop species, crop S status, and fertilizer regime/timing (Stroud et al., 2010).

For Se(IV), S fertilizers mostly produce competitive inhibition vis-a-vis the absorption of Se(IV). Mackowiak and Amacher (2008) showed that S fertilizer application reduced Se(IV) uptake by plants such as alfalfa and wheat grass. Dhillon and Dhillon (2000) reported that the application of 0.8 t hm^–2^ gypsum to selenite-rich soil significantly decreased total Se accumulation in rice, while increasing S content, with minimal reductions in Se concentration in grains and straw by 35 and 36%, respectively. S application can significantly inhibit Se(IV) absorption and accumulation (Lee et al., 2011; Liu et al., 2016a), but a hydroponic experiment also showed that selenite and sulfate had no antagonistic effects on Se uptake (Li et al., 2008). This suggests that the inhibitory effect of S fertilizer on Se(IV) uptake and accumulation by plants under soil cultivation depends on other processes rather than competitive adsorption. S fertilizer application to the soil may change the physical and chemical behavior of Se(IV) in the soil. For example, S fertilizer significantly increases soil organic matter content, significantly decreases soil pH, and increases microorganismal activity, which, in turn, may increase the amount of Se(IV) fixed in soil (Liu et al., 2015). Therefore, on Se-deficient soils with rich organic matter content and sufficient S, Se uptake by crops can be increased by reduced S fertilizer application. On the other hand, for Se-rich soils, organic matter deficiency, and insufficient S, it is possible to reduce Se uptake by crops via increased S-fertilizer and organic-fertilizer application. S-fertilizer and organic-fertilizer management is therefore a critical determinant of Se accumulation in the crop.

## Se Interaction With Heavy Metals (As, Hg, and Cd)

### The Role of Selenium in the Regulation of Arsenic Bioavailability and Accumulation

As is a highly toxic environmental pollutant, whose pollution is widespread throughout the world, including in many parts of United States, Europe, China, and Southeast Asia (Rahman et al., 2014). As and Se have a relatively close positioning in the periodic table of elements, and both are metalloids (even though As is often classified as a “heavy metal”), with similar chemical properties, and one can infer similar characteristics in plant absorption as well as in transport from the soil to plants. In recent years, in attempts to clarify the antagonistic effects Se and As have on one another, much insightful research has been produced. It is now clear that the antagonistic effects of As and Se on one another depend on the chemical forms of Se and As, the levels of Se and As in soil, and plant genotype (Table 3).

**TABLE 3:** Research progress on the effects of Se on accumulation of heavy metals in higher plants.

Added Se form	Amounts	Heavy metal species	Crop species	Test results	Heavy metal availability	References
Se(IV)	0, 0.5, 1 mg kg^–1^	As	Rice	The addition of Se (IV) reduced total As content in soil solution, but increased As content in rice grain by 43.7–74.6%.	Decrease	Wan et al., 2018
Se(IV)	0, 1, 5, 10 mg kg^–1^	As	Rice	Se (IV) application significantly reduced rice grain As content by 8.6, 31.4, and 33.7% with the Se content of 1, 5, and 10 mg kg^–1^, respectively.	–	Zhou et al., 2017b
Se(IV)	0, 1, 5 mg kg^–1^	As	Rice	Se (IV) application reduced As availability in rhizosphere soil. Se (IV) application significantly reduced the grain As content.	Decrease	Lv et al., 2020
Se(IV)	0, 0.5, 3, 6 mg kg^–1^	Hg	Rice	Se(IV) addition forms IHg-Se complexes, resulting reduction of effectiveness of Hg in soil. As content in rice grain reduced with the increase of Se (IV) concentration.	Decrease	Tang et al., 2017
Se(IV)	0, 20, 40, 60, 100, 300, 500 mg kg^–1^	Hg	Rice	Se (IV) application decreased inorganic Hg and MeHg concentration in grain and root.	Decrease	Xu et al., 2019
Se(IV)	0, 3 mg kg^–1^	Hg	Rice	Se(IV) application reduced MeHg content in soil and in rice root, straw and grain.	Decrease	Wang et al., 2016
Se(IV)	1 mg kg^–1^	Cd	Rice	Se(IV) reduced exchangeable Cd content but increased the Cd content combined with carbonate and iron-manganese oxides. Se(IV) addition reduced rice Cd content by 36.5%	Decrease	Huang et al., 2018
Se(IV)	0, 0.5, 1, 2, 4, 8 mg kg^–1^	Cd	Wheat	Se(IV) reduced Cd content in wheat shoot from 25 to 35%.	–	Atarodi et al., 2018
Se(IV)	0, 1, 5, 10, 15, 20 mg kg^–1^	Cd	Rape	Se(IV) markedly reduced Cd concentration in both root and shoot.	–	Wu et al., 2016b
Se(IV)	0, 1, 5 mg kg^–1^	Cd	Rice	Se (IV) application reduced Cd availability in rhizosphere soil. Se (IV) application significantly reduced the grain Cd content.	Decrease	Lv et al., 2020
Se(VI)	0, 0.1, 1, 5 mg kg^–1^	As	Rice	Se(VI) addition reduced As content in rice root, shoot and grain.	–	Liao et al., 2016
Se(VI)	0, 0.5, 3, 6 mg kg^–1^	Hg	Rice	Se(VI) addition forms IHg-Se complexes, resulting reduction of effectiveness of Hg in soil. Hg content in rice grain reduced with the increase of Se (VI) concentration.	Decrease	Tang et al., 2017
Se(VI)	0, 0.1, 1, 5 mg kg^–1^	Cd	Rice	Se(VI) addition reduced Cd content in rice root, shoot and grain.	–	Liao et al., 2016
Se(VI)	0, 3 mg kg^–1^	Hg	Rice	Se(VI) application reduced MeHg content in soil and in rice root, straw and grain.	Decrease	Wang et al., 2016
Undefined	0, 0.003, 0.03, 0.15, 0.3, 1.5 mg kg^–1^	Hg	Rice	Low Se concentration reduced Hg and MeHg content in rice grains, but high Se concentration increased it.	–	Li et al., 2019a

First, As(III) and Se(IV) enter the plant root system by sharing the same transporter, and there is competitive interaction between them at the influx level (Ma et al., 2008; Zhao X. et al., 2010; Li N. et al., 2016). That Se reduces As transport to the shoot has been confirmed in many studies (Chauhan et al., 2017; Camara et al., 2018). However, Bluemlein et al. (2009) reported that As(III) and Se(IV) have a synergistic effect in rice roots. This phenomenon has also been reported in other crops (Han et al., 2015). The exact mechanism is not clear, but this phenomenon is very interesting and needs further study.

The addition of Se significantly limits the transport of As from the root to the shoot. Hu et al. (2013) found that Se(IV) can significantly inhibit the transport of both As(III) and As(V) from root to shoot in rice. Zhou et al. (2017a) showed that Se can reduce the transport of As from soil to root, then from root to shoot and then to the grain in rice. Se restricts the transport of As for two main reasons: (1) As(V) absorbed by plants is reduced to As(III) in root cells, and As(III) can be chelated in a Lewis acid-base reaction by reduced glutathione (GSH) or by phytochelatins (PCs) and finally stored in the root vacuole, thus curtailing As transport from root to shoot (Ye et al., 2011). Liao et al. (2016) showed that Se(IV) and Se(VI) application to the soil can reduce As concentration in the grains and husks. At the same time, Se(IV) was more effective than Se(VI) at reducing the grain As concentration in rice. (2) Se affects As transport direction at the stem node and transport in the phloem from leaves to grains. As is strongly accumulated in the vascular bundle stem node. The stem node is a central point of translocation from the xylem to the phloem, which plays a critical role in As distribution (Moore et al., 2014; Wan et al., 2018). The mechanism may be linked to enhanced development of apoplastic barriers (Casparian bands and suberin lamellae) in the endodermis by Se, which would limit the translocation of heavy metals into the xylem and then the shoots more broadly (Wang X. et al., 2014). This important connection of Se supplementation to barrier formation in roots and at the root-shoot interface requires further study, also with a view to water-use efficiency in plants (Plett et al., 2020).

**FIGURE 2:**
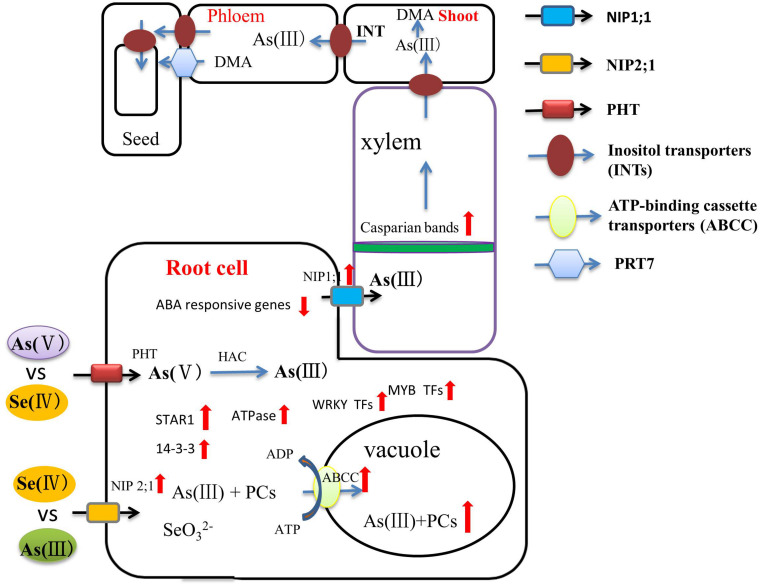
A flow diagram of the role of Se in regulating As bioavailability and accumulation and transport through xylem and phloem to the grains. Inositol transporters (INTs) may be involved in the mobilization of As from source tissues to the phloem for subsequent transport to seed tissues. As(III) and Se(IV) enter the plant root system by sharing the NIP2;1 transporter. As(V) and Se(IV) are absorbed by plant roots via phosphate transporters. ABC transporters can sequester As(III)-PCs complexes into vacuoles. Se significantly limits the transport of As from the root to the grain through enhanced development of apoplastic barriers and up-regulation of related genes (red arrow).

Recently, several studies have addressed Se-mediated improvement of As toxicity from the perspective of genomics and proteomics. Transporters are the pivotal proteins that regulate As in above-ground parts of the plant. OsPTR7, a putative peptide transporter is involved in the long-distance translocation of DMA and contributes to the accumulation of DMA in rice grain (Tang et al., 2017). Inositol transporters (INTs) are responsible for As(III) loading into the phloem, the key source of arsenic in seeds (Duan et al., 2016). Se can regulate the expression of these transporter proteins and, thus, directly affect As accumulation in the above-ground parts. Song et al. (2014) found that ABC transporters can sequester As(III)-PCs complexes into vacuoles in both *Arabidopsis* and rice. Chauhan et al. (2020) showed that the up-regulation of ABC transporters, ATPases, STAR1 protein, and 14-3-3 protein during As and Se co-exposure on both the transcriptome and proteome levels, some of which are also effective in limiting aluminum toxicity, indicating that Se can influence a reduction of As accumulation in rice by these means of facilitated vacuolar sequestration. The higher expression of regulation of WRKY, AUX/IAA, and MYB transcription factors that link to As tolerance and reduced expression of phosphate transporters, reducing As uptake, during As and Se co-exposure were also observed (Figure 2). However, detailed molecular analysis is needed to clarify the As/Se interactions in different plants further. This is of particular pressing important in rice, where As accumulation in the grain, in many common rice-cultivation areas, poses a significant health hazard to humans (Rahman and Hasegawa, 2011).

In the future, genomic and proteomic analyses will need to be combined to decipher with precision the molecular mechanisms by which Se reduces plant-shoot As accumulation, thus laying a foundation for comprehensively and thoroughly mapping out the molecular network of As regulation by Se.

### The Role of Selenium in the Regulation of Mercury Bioavailability and Accumulation

Mercury (Hg) is a global pollutant and carries significant toxicity to human health at very low concentration of exposure (Wang et al., 2016). Methyl mercury (MeHg) can be generated from inorganic Hg (IHg) by anaerobic microorganisms under flooded conditions. This form is then easily absorbed by plant roots, rapidly transported to edible plant parts, and, thereby, can directly threaten human health. Indeed, food consumption is the main source of MeHg exposure to humans (Zhang et al., 2010). As in the case of arsenic accumulation in the grain (see above), the problem of excessive concentration of MeHg in rice grains needs urgent attention. Soil Hg remediation technologies typically have the shortcomings of high cost and negative impact on the soil. However, recent studies have shown that Se can inhibit the transport and accumulation of Hg in plants, and this connection, therefore, must be understood (Table 3).

**FIGURE 3:**
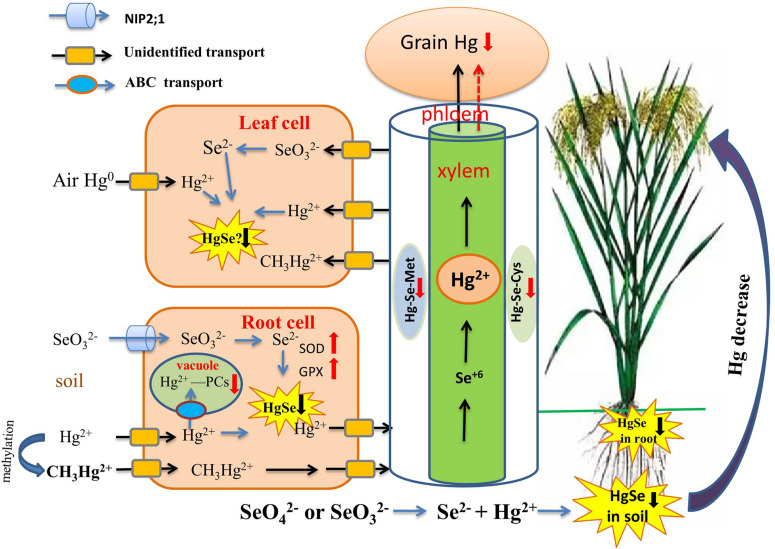
Overview of the role of Se in regulating Hg bioavailability and accumulation. Se reduces Hg transport from the root to the shoot, and then to the grain through the formation of insoluble HgSe in soil, roots, and possibly leaves (black down arrow). Then Hg-PCs complexes are sequestered in vacuoles and phloem, which are restricted in their upward Hg migration (red down arrow). Se reduces Hg uptake by plants by elevating the activities of SOD, POD, CST, GSH-Px, and GPX (red up arrow).

The antagonism of Se-Hg in the soil is a key process controlling Hg behavior in the soil-rice system (Figure 3). Recently, a series of studies by Wang X. et al. (2014); Wang et al. (2016) have provided a definitive explanation for the mechanism of antagonism of Se-Hg in soil. Se(IV) and Se(VI) were equally effective in reducing soil MeHg concentrations, possibly because of rapid changes in Se speciation and direct chemical reaction with Hg. Several recent experimental confirm the importance of the Se-Hg interaction in the soil Xu et al. (2019), at least for rice:

(i) A direct chemical reaction between Se and Hg involves several steps and affects Hg bioavailability: Se(IV) and Se(VI) can be reduced to Se^2–^ and Se^0^ through microbial metabolism. Se^2–^ and Hg^2+^ then react directly and produce the insoluble compound HgSe, thereby achieving sedimentation and inhibiting Hg methylation. The reaction formula is as follows:

(1) $$Hg^{2+}+Se^{2-}=HgSe\text{or}Hg^{0}+Se^{0}=HgSe$$

Since the solubility constant for the reaction product (10^–58^), HgSe, is much smaller than that of HgS (10^–52^), Hg^2+^ preferentially reacts with Se^2–^ ions to produce HgSe complex precipitate. Therefore, compared with S^2–^ (HS^–^), Se^2–^ (HSe^–^) is more likely to react with Hg^2+^ and will form HgSe instead of HgS. TEM-EDX analysis results revealed that HgSe nanoparticles were formed in Se-applied soil in an anaerobic environment, which provided strong evidence for the direct Se-Hg interaction in soil (Wang et al., 2016). In addition, Chang et al. (2020) suggested that rice leaves can directly take up elemental Hg from the atmosphere, and Hg-Se complex may then be formed in rice leaves.
(ii) Co-exposure of Hg and Se can inhibit the growth of the sulfate-reducing bacterium D. *desulfuricans* when compared to Hg exposure alone (Truong et al., 2013), and this step is critical for the methylation of IHg, leading to reduced production of MeHg;
(iii) Se addition also increases demethylation and evaporation of MeHg, resulting in decreased production of soil MeHg (Dang et al., 2019).

Se-Hg antagonism can also occur in dryland ecosystems, the mechanism for which is also that formation of insoluble HgSe complexes that precipitate and reduce Hg bioavailability and absorption by dryland plants. Tran et al. (2018a) demonstrated that the reduction in bioavailability of Hg depends on Se species; Se(IV) was more effective than Se(VI) in limiting Hg bioavailability and absorption, for pakchoi planted in dryland soil. It needs to be noted that such an effect only manifests significantly when Se(IV) and Hg are at a level of 2.5 and 3.0 mg kg^–1^, respectively.

In addition to the formation of insoluble complexes, Hg-Se antagonism also plays out on a different, plant-internal, level. The detoxification of Hg, once absorbed, occurs by regulation of the metabolic production of antioxidant and chelation compounds in plants, which, in turn, presents an additional strategy for remediation of soil Hg and potentially reducing nutritional exposure to Hg if resultant compounds do not enter edible plant tissues. Tran et al. (2018b) showed that the Se application at 1.0 and 2.5 mg/kg led to significant reductions on Hg uptake by plants and increases in growth of pakchoi by elevating the activities of superoxide dismutase (SOD), peroxidase (POD), catalase (CST),glutathione peroxidase (GSH-Px) enzymes. In addition, Diao et al. (2014) found that Se could significantly increase GPX gene expression and enzyme activity. Third, Se can increase rice apoplastic barriers in the endodermis, which hinder both IHg and MeHg uptake (Zhou et al., 2014, 2017b; Wang X. et al., 2014). In addition, after Se treatment, there is less Hg in rice tissue due to the increased amount of iron plaque on the root surface, a natural barrier that prevents the accumulation of metal(loid)s in plants (Li Y. et al., 2016; Zhou and Li, 2019; Huang G. et al., 2020).

Selenium (Se) can also reduce Hg distribution in the embryo and endosperm of grains (Meng et al., 2014), which is very important for reducing the risk of human exposure to Hg. Despite the recent significant research progress in the Hg-Se interaction in plants, the mechanism by which Se reduces bioavailability of Hg in soil-plant systems has not yet been fully elucidated, and further research is needed to explore the effects of Se on Hg-methylated anaerobic microorganisms as well as the mechanism of Se-Hg antagonism. There is a need to conduct in-depth studies on the mechanism by which Se promotes Hg detoxification, both before it enters the plant and thereafter, and the mechanism by which Se prevents Hg entry into the grain, which carries important practical significance for effectively lowering the risk of human exposure to Hg while addressing the problem of insufficient dietary Se intake.

### The Role of Selenium in the Regulation of Cadmium Bioavailability and Accumulation

Cd in the soil tends to accumulate in the roots of crops, followed by ready transport to edible parts and seriously affect human health (Hamid et al., 2019). Se application is considered as a potential solution to reduce Cd accumulation in plants. Indeed, the antagonism between Se and Cd has been studied in many different plants (Wan et al., 2016; Wu et al., 2016a; Affholder et al., 2019).

Antagonism between Se and Cd in the soil is complex and depends on factors such as Se and Cd dosage, applied Se species, and crop genotype (Table 3). Shanker et al. (1995) proposed that Se and Cd form CdSe complexes in soil unusable by plant roots. Huang et al. (2018) showed that Se can decrease total Cd concentration in soil solution by an average of 11.2–13.0%, eventually affecting Cd uptake by plants. The mechanism has yet to be verified. Cd-Se antagonism also depends strongly on the prevailing Se species in soil solution. Liao et al. (2016) reported that Se(IV) can lower rice grain Cd concentration more effectively than Se(VI). This might be because Se(IV) has smaller mobility than Se(VI) in soil, leading to facilitated Cd-Se complexation and in reduced Cd absorption. Se-Cd antagonism also relates to Se and Cd dosage. Zhao et al. (2019) reported that, at 1 mmol L^–1^ Cd, there was no significant difference in Cd concentration in the shoot and root of oilseed rape treated with Se at various levels; at 5 mmol L^–1^ Cd, however, Se addition (at 0.1 and 1 mmol L^–1^) significantly decreased root and shoot Cd concentration. Se addition (at 10 mmol L^–1^) significantly increased the concentration in both organs. It is possible that excessively high Se dosage damages rape seedlings (Saidi et al., 2014). Ding et al. (2014) reported that a high dose of Se increased the permeability of the root cells to Cd, related to damage to the root cell membrane at elevated Cd concentrations. Hence, applying a well-adjudicated dose of Se, calibrated to the soil and crop system in question, is critical. By summarizing all the published studies on Se-Cd antagonism in plants to date Affholder et al. (2019) derived a quantitative model for predicting Se-Cd antagonism. Based on the model calculations, the authors concluded that, when the amount of Se applied to the root medium was 0.013, 0.082, and 142 mg Se kg^–1^, crop Cd concentration was decreased by 10, 25, and 50%, respectively.

**FIGURE 4:**
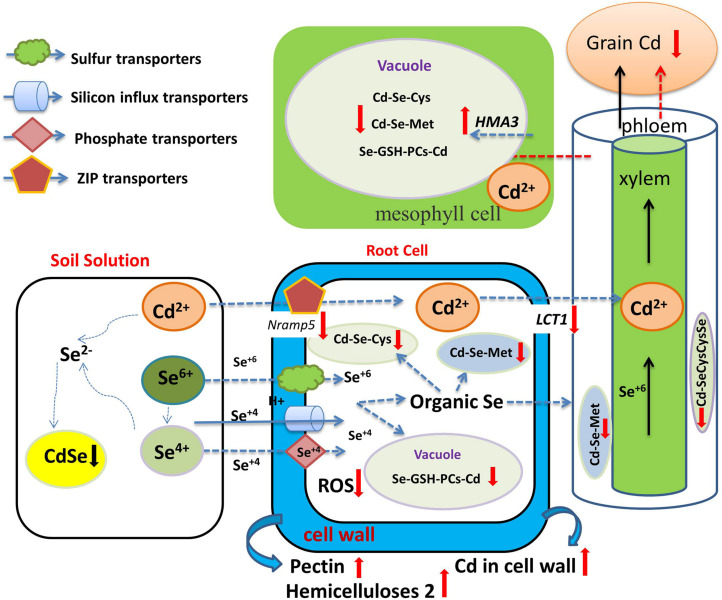
Overview of the role of Se in regulating Cd bioavailability and accumulation. Se and Cd form CdSe complexes in soil unusable by plant roots (black down arrow). Se-Cd antagonism not only occurs in soil, but also manifests inside plant cells through the formation of Se- and Cd compounds, namely Cd-SeCysCysSe and Cd-SeMet (red down arrow), which are restricted in their upward, shoot-bound, Cd migration. Se reduces the expression of genes coding for Cd uptake and of transport-related genes (red down arrow), activates the expression of the tonoplast-resident transporter gene (red up arrow) and increases Cd accumulation in the plant cell wall (red up arrow). Se also increases GSH and PCs and reduces Cd transport from the root to the shoot and to the grain.

Se-Cd antagonism not only occurs in soil, but also manifests inside plant cells. Huang et al. (2018) reported that Se application significantly reduced Cd concentration of different tissues and the translocation of Cd from roots to shoots in mature rice. However, Chang et al. (2013) reported that Se addition did not affect Cd concentration in rice roots, but reduced Cd accumulation in stems, leaves, rice husks, and grains. Pedrero et al. (2008) reported that Se addition decreased Cd concentration of the edible parts in broccoli, but increased Cd concentration in roots. These results indicate that the mechanism by which Se reduces the accumulation of Cd in aboveground tissue may be in the enhancement of root sequestration and the reduction of root-to-stem transport. Studies have shown that Cd and Se are absorbed by plant roots through different pathways or transporter proteins. Cd is absorbed by plant roots through zinc transfer proteins (ZIP) or cation channels (Lux et al., 2011), while Se is absorbed by plants through sulfate and phosphate transporters (Zhang L. et al., 2014, and see above). Se and Cd are absorbed by different transporters in the plant root system, so there is no direct competitive absorption relationship between Se and Cd. Recent years have witnessed great progress in our understanding of the mechanism of Se-Cd antagonism. Wu et al. (2016b) reported that Se application had no effect on Cd concentration or ratio in root saps, and Wan et al. (2016) showed that Se(IV) only played a minor role in Cd influx into rice roots, while Se(VI) had no effect at all. These results indicate that there is no competitive relationship in Se and Cd at the primary absorption level in plant roots. Se appears to only reduce Cd translocation from the root to the shoot, and a major component of this is the increase in GSH and PCs in the plant when Se supplementation is in play (see as well above). Cd is easily bound by S-containing ligands, such as those present in phytochelatins (PCs), metallothioneins, and glutathione (GSH) (Yadav, 2010). Yu et al. (2019) showed that the relative proportion of SeMet and Se(VI) was the main factor in the regulation of Cd accumulation in the plant. Zhang (2019) further showed that Se- and Cd-binding compounds, namely Cd-SeCysCysSe and Cd-SeMet, appeared in the stalk and leaf tissues after Se application in corn. These compounds are restricted in their upward, shoot-bound migration of Cd in plants (Figure 4).

One of the main effects of Cd toxicity is the inhibition of photosynthesis in higher plants (Wang Y. et al., 2014; Ahmad et al., 2016). Se application has been shown to reduce ethylene content, promote the accumulation of proline, and increase the activities of glutathione reductase (GR) and glutathione peroxidase (GPX) in wheat under Cd stress (Khan et al., 2015).

Zhao et al. (2019) found that Se increased the contents of pectin and hemicellulose 2. By combining with Cd ions, these compounds can limit Cd transport across cell plasma membranes, thereby reducing the entry of Cd into the roots and its transport to stems, pods, and seeds. Cui et al. (2018) reported that Se application reduced the expression of genes coding for Cd uptake (*OsNramp5*) and transport-related genes (*OsLCT1)*, and reduced Cd uptake by rice cells, but activated the expression in the tonoplast transport gene *OsHMA3* and lignin synthesis (*OsPAL, OsCoMT Os4CL3*)-related genes, making it more difficult for Cd to be transported from the vacuole to the phloem, and increasing Cd accumulation in the plant cell wall.

In summary, Se biofortification and reducing the risk of Cd pollution may have dual benefits. However, the results of the interactions between Se and Cd mainly depend on the rate of applications, plant species, and soil conditions (Feng et al., 2013; Wan et al., 2016). Under the context of high Cd, Se-Cd interaction may be another case, and more studies are needed. Yang et al. (2019) concluded, based on field experiments, that high-Se soil did not reduce the absorption of Cd in rice, and high Se helped increase Cd translocation from root to stem. The reason may be that excessive Cd stress in this case may have caused serious damage to membranes and cell walls, chloroplasts, and other cell structures, resulting in decreased cellular integrity, photosynthetic rate, and plant growth. As well, Se application cannot reverse Cd damage to plant cells already incurred (Alves et al., 2020). Feng et al. (2013) found that Se increased the absorption of Cd and, when the Cd concentration was extremely high, deleterious effects on rice growth were readily evident. The authors linked this finding to cell membrane damage caused by Se application, increasing the permeability of root cells to Cd (Feng et al., 2017). This serves as an important warning that, in actual crop production settings, Cd reduction by Se application cannot automatically be assumed and requires more tests to determine precise application conditions, and blind implementation cannot be recommended.

## Conclusion and Future Perspectives

In this review, we elucidate the nutritional effects of Se in plants and their consequences for crop consumption by humans, and summarize the basic mechanisms of uptake, long-distance transport, and biofortification of Se in grains. We further discuss Se biofortification and interaction mechanisms with the macronutrient elements N, P, and S and the heavy metals Hg, As, and Cd. For human nutrition, any ideal crop must contain essential mineral elements such as Se, coupled to minimized accumulation of toxic elements in all edible parts, which are, typically, above-ground tissues. However, to achieve this goal, several hurdles need to be overcome:

(1) A comprehensive understanding of the uptake and transport systems for Se is required. Uptake and transport of Se from soils to edible plant parts is mediated by various transporters. However, we are far from fully understanding the molecular mechanisms of long-distance transport of Se to edible parts. Many transporters for Se remain to be identified. It is necessary to exploit the transporters regulating the primary absorption, internal transport, and accumulation of Se in plants, and transfer genes that code for them to relevant target plants to effect efficient expression, using transgenic technology or gene editing technology to increase the concentration of Se in non-Se-rich plants.
  From the perspective of human nutrition and health, currently, it is urgent not only to increase the concentration of Se in the edible parts of crops, but also to increase some specific chemical Se forms, as different Se forms have varying nutritional value and toxicological properties in the human body. For instance, SeMeSeCys has promising anti-cancer properties, and overexpression of SeCys methyltransferase (SMT) may therefore present as the best choice for biofortification with a view to human nutrition. Applying modern techniques of molecular biology, targeted genes may also be overexpressed in specific plant tissues, such as in cereal reproductive structures, or in vegetative tissues of plants from which anticarcinogenic compounds can be easily extracted for large-scale production, such as in *Medicago* or in vegetable crops.
(2) It is important to further examine the effects of nutrient fertilizers (N, P, S, but also K and Ca/Mg) on plants in intensive agriculture production systems, especially vis-a-vis their effects on Se bioconcentration in grains, investigate the physiological and molecular mechanisms by which macronutrients affect long-distance transport of Se in crops, define agronomic production measures that simultaneously meet the high-yield nutritional needs of crops and nutritional balance requirements for Se, thus providing the theoretical and technical support needed for the genetic improvement of Se-rich crops and human health.
(3) The interaction mechanism between Se and heavy metals remains understudied. It is necessary to strengthen the research on the mechanisms by which Se may antagonize the toxicity of heavy metals, and study the effects of Se on the migration and transformation of heavy metals in soil-crop systems, especially the mechanisms by which Se restricts long-distance transport of heavy metals in cereal crops to the grain. It is urgent to conduct in-depth studies on the interactions between Se and heavy metals by using in-situ analytical techniques, such as synchrotron imaging. At the same time, a large number of field tests on different soil types are needed to clarify the effect of Se in improving Se nutrition level in crops and reducing heavy metal concentration in crops as well as refining the application conditions in a manner that might make them amenable to the application of precision agriculture techniques vis-a-vis Se. This will provide reference for using Se as a biostimulant to increase crop yields, improve crop nutritional value, and reduce the toxicities of soil metal toxicants.

## Author Contributions

XZ and JY wrote, drafted and checked the manuscript. WS designed and executed the manuscript. HK co-wrote the manuscript. All authors contributed to the article and approved the submitted version.

## Conflict of Interest

The authors declare that the research was conducted in the absence of any commercial or financial relationships that could be construed as a potential conflict of interest.
